# Enhanced Photocatalytic Properties and Photoinduced Crystallization of TiO_2_–Fe_2_O_3_ Inverse Opals Fabricated by Atomic Layer Deposition

**DOI:** 10.1021/acsami.4c10831

**Published:** 2024-09-03

**Authors:** Carina Hedrich, Nithin T. James, Laura G. Maragno, Valéria de Lima, Sergio Yesid Gómez González, Robert H. Blick, Robert Zierold, Kaline P. Furlan

**Affiliations:** † Center for Hybrid Nanostructures, Universität Hamburg, 22761 Hamburg, Germany; ‡ Hamburg University of Technology (TUHH), Institute of Advanced Ceramics, Integrated Materials Systems Group, Denickestraße 15, 21073 Hamburg, Germany; § Federal University of Santa Catarina (UFSC), Department of Chemical and Food Engineering (EQA), 88040-970 Florianópolis, SC, Brazil

**Keywords:** atomic layer deposition, inverse opal, photocatalysis, photoinduced crystallization, semiconductor heterostructure, multilayer thin films

## Abstract

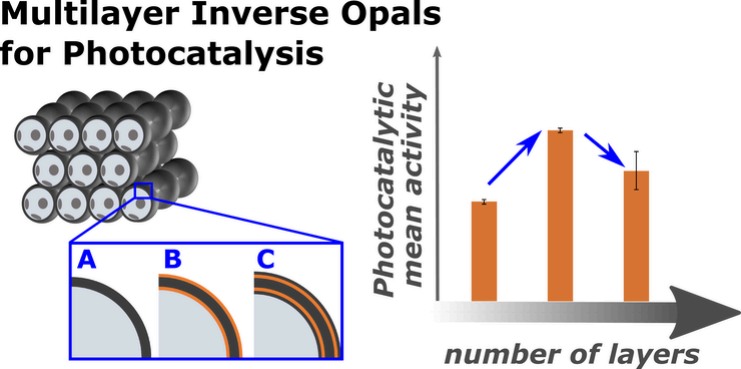

The use of solar energy for photocatalysis holds great potential for sustainable pollution reduction. Titanium dioxide (TiO_2_) is a benchmark material, effective under ultraviolet light but limited in visible light utilization, restricting its application in solar-driven photocatalysis. Previous studies have shown that semiconductor heterojunctions and nanostructuring can broaden the TiO_2_’s photocatalytic spectral range. Semiconductor heterojunctions are interfaces formed between two different semiconductor materials that can be engineered. Especially, type II heterojunctions facilitate charge separation, and they can be obtained by combining TiO_2_ with, for example, iron(III) oxide (Fe_2_O_3_). Nanostructuring in the form of 3D inverse opals (IOs) demonstrated increased TiO_2_ light absorption efficiency of the material, by tailoring light-matter interactions through their photonic crystal structure and specifically their photonic stopband, which can give rise to a slow photon effect. Such effect is hypothesized to enhance the generation of free charges. This work focuses on the above-described effects simultaneously, through the synthesis of TiO_2_–Fe_2_O_3_ IOs via multilayer atomic layer deposition (ALD) and the characterization of their photocatalytic activities. Our results reveal that the complete functionalization of TiO_2_ IOs with Fe_2_O_3_ increases the photocatalytic activity through the slow photon effect and semiconductor heterojunction formation. We systematically explore the influence of Fe_2_O_3_ thickness on photocatalytic performance, and a maximum photocatalytic rate constant of 1.38 ± 0.09 h^–1^ is observed for a 252 nm template TiO_2_–Fe_2_O_3_ bilayer IO consisting of 16 nm TiO_2_ and 2 nm Fe_2_O_3_. Further tailoring the performance by overcoating with additional TiO_2_ layers enhances photoinduced crystallization and tunes photocatalytic properties. These findings highlight the potential of TiO_2_–Fe_2_O_3_ IOs for efficient water pollutant removal and the importance of precise nanostructuring and heterojunction engineering in advancing photocatalytic technologies.

## 1 Introduction

Solar-driven photocatalysis has emerged as a promising self-sustainable technology for removing water pollutants by harnessing solar energy to decompose organic contaminants.^1−3^ Among various photocatalysts, titanium dioxide (TiO_2_) is a benchmark material based on its excellent chemical stability, biocompatibility, and photocatalytic activity under ultraviolet (UV) light irradiation.^4−7^ However, its wide band gap prevents the utilization of visible light, which constitutes the majority of the sunlight spectrum, and thereby limits its practical applications. In recent years, efforts have been made to improve the light harvesting of TiO_2_ by various strategies, such as doping with other elements, formation of semiconductor heterostructures, or nanostructuring of the material.^6−8^ The latter approach is based on increasing the surface area and light trapping in such structures.

Inverse opals (IOs) are an example of a nanostructured material characterized by a periodically ordered porous structure. They offer the possibility to tune light-matter interactions within the structure based on their photonic crystal (PhC) structure.^9,10^ PhCs feature so-called photonic stopbands (PSBs), i.e., spectral regions in which light of the respective wavelength cannot propagate through the structure and thus, the light is reflected by the PhC three-dimensional (3D) structure.^9,11^ The PSB position is determined by the composition and geometry of the PhC, namely the refractive indices of the utilized materials and the structural parameters, such as template size and spacing.^9,12,13^ Hence, modifications of the IO’s structural parameters enable the tuning of the PSB position and allow, for instance, to position it across the whole UV to infrared range. Note, the group velocity of photons inside a PhC is strongly reduced at the PSB edges due to the slow photon effect.^14,15^ This effect leads to an increment of the interaction probability of photons with the PhC material.

Consequently, the generation of free charge carriers by absorption of photons in a semiconductor photocatalyst nanostructured within an IO 3D structure can be enhanced when the material’s electronic band gap is aligned with the PhC’s PSB edge and, thus, potentially boost its’ photocatalytic performance. Another approach to further improve the activity of a photocatalyst is to facilitate charge separation. Combining different semiconductors results in heterojunctions at the interfaces, which tune the migration of free charge carriers through the structure.^8,16^ Specifically, type II heterojunctions of two semiconductors direct electrons (e^–^) and holes (h^+^) to the different materials, thereby, separating them and reducing their recombination. For example, iron(III) oxide (Fe_2_O_3_) as a visible light-active semiconductor photocatalyst can be combined with TiO_2_ to improve charge separation and to widen the light absorption range, potentially leading to a further increase in photocatalytic performance. Fe_2_O_3_ is abundant on Earth, cheap, and nontoxic, rendering it a promising candidate for photocatalytic applications.^17,18^ However, inherent limitations such as inefficient charge carrier generation or fast recombination of photogenerated charge carriers need to be overcome.^19−21^ Previous reports about TiO_2_–Fe_2_O_3_ heterostructure thin films and Fe_2_O_3_ coated TiO_2_ nanostructures prove the concept of enhancing the photocatalytic properties by adding Fe_2_O_3_ as visible light absorbing material to TiO_2_ due to semiconductor heterojunction formation.^19,22−32^

Moreover, Liu et al. and Pylarinou et al. reported further improvement of the photocatalytic activity of TiO_2_ IOs when decorating them with Fe_2_O_3_ nanoparticles or nanoclusters, respectively, based on the slow photon effect when the PSB edge is aligned with the Fe_2_O_3_ band gap.^33,34^ Liu et al. synthesized the nanoparticles at the TiO_2_ IOs by a hydrothermal method, while Pylarinou et al. utilized a chemisorption-calcination-cycle technique to deposit Fe_2_O_3_ nanoclusters.^33,34^ However, coating TiO_2_ IOs with Fe_2_O_3_ films to encapsulate the complete TiO_2_ film has not been reported yet. Such structure could affect the photocatalytic performance because only either Fe_2_O_3_ or TiO_2_ is in contact with the environment, thereby further increasing the importance of charge carrier separation. Since Fe_2_O_3_ often suffers from a short hole diffusion length of only a few nanometers and, thus, limited charge carrier separation, precise control over the film thickness is essential.^6,18^ Besides forming semiconductor heterojunctions to facilitate charge separation, the fabrication of Fe_2_O_3_ thin films by atomic layer deposition (ALD) allows for very defined thicknesses based on the self-limiting reactions during the ALD process.^35^ Hence, the Fe_2_O_3_ film thickness can be optimized for maximum photocatalytic performance.

Here, we report on the synthesis of TiO_2_–Fe_2_O_3_ multilayer inverse opals by ALD and assessment of their photocatalytic properties. We demonstrate that the complete functionalization of TiO_2_ IOs with Fe_2_O_3_ by ALD enhances their photocatalytic properties by concomitantly forming semiconductor heterojunctions (material combination) and activating the slow photon effect (nanostructuring into IOs). In addition, the influence of the Fe_2_O_3_ thickness on the photocatalytic performance of TiO_2_–Fe_2_O_3_ bilayer IOs is studied to further improve the efficient utilization of photogenerated charge carriers. Moreover, TiO_2_–Fe_2_O_3_ IOs are overcoated with another TiO_2_ thin film by ALD to investigate the effect on the photocatalytic performance. These TiO_2_–Fe_2_O_3_–TiO_2_ multilayer IOs exhibit reduced photocatalytic activities compared to the bilayer IOs due to nonoptimal heterojunction configuration leading to the charge carriers’ trapping. However, the TiO_2_–Fe_2_O_3_–TiO_2_ multilayer IOs provoke photoinduced crystallization of the amorphous TiO_2_ layers to anatase, which enhances their photocatalytic properties.

## 2 Experimental Section

### 2.1 Materials

Mucasol solution was purchased from Brand GmbH (Germany), and 5 w/v% aqueous polystyrene (PS) particles’ dispersions with particle sizes of 150 ± 3 nm and 252 ± 6 nm were acquired from microParticles GmbH (Germany). Ultrapure “Milli-Q” water (>16 MΩ cm, H_2_O) was utilized as oxidant precursor for the ALD cycles and to prepare aqueous dispersions for colloidal self-assembly performed on borosilicate glass cover slides from Paul Marienfeld GmbH (Germany). Methylene blue (C_16_H_18_ClN_3_S, MB, CAS 122965–43–9), and hydrogen peroxide (H_2_O_2_, CAS 7722–84–1) were supplied by Sigma-Aldrich (Germany), while titanium tetraisopropoxide (TTIP, CAS 546–68–9) and ferrocene (C_10_H_10_Fe, Cp_2_Fe, CAS 102–54–5) were purchased from Alfa Aesar (Germany). Nitrogen (6.0) was received from SOL (Germany), and oxygen (5.0) was supplied by Westfalen (Germany), respectively.

### 2.2 Fabrication of TiO_2_–Fe_2_O_3_ Inverse Opals

**Figure 1:**
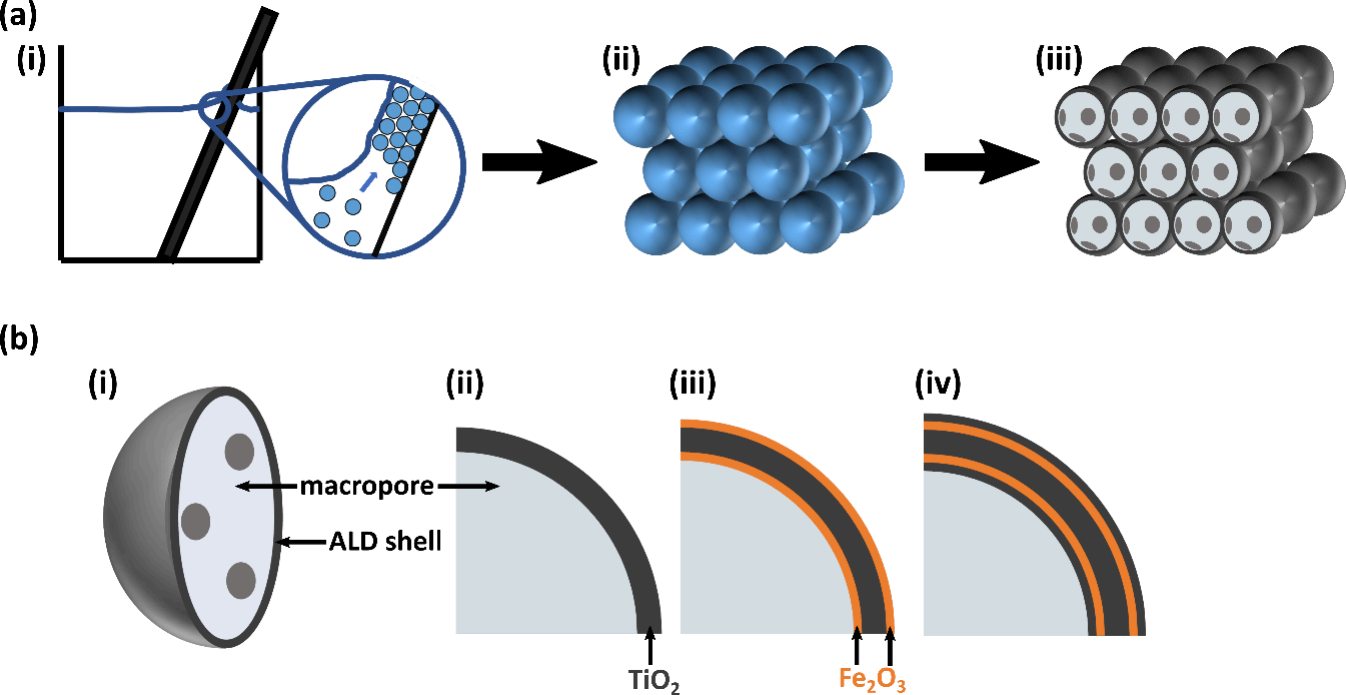
Schematic drawing of the fabrication of TiO_2_–Fe_2_O_3_ IOs and their shell composition. (a) Different steps in the fabrication process show (i) self-assembly of PS spheres, (ii) an assembled PS sphere opal template, and (iii) a TiO_2_ inverse opal after ALD coating and burn-out of the polymer template. The latter scheme presents cuts through the front row of spheres to visualize the hollow inside and gaps connecting neighboring macropores. (b) The TiO_2_ IO structure presented in (i) and (ii) is further modified by ALD functionalization to produce (iii) TiO_2_–Fe_2_O_3_ bilayer IOs and (iv) TiO_2_–Fe_2_O_3_–TiO_2_ multilayer IOs.

Preparation of TiO_2_–Fe_2_O_3_ IOs starts with the colloidal self-assembly of PS particles, followed by coating of the self-assembled direct opal structures with TiO_2_ by ALD, removal of the PS template, functionalization with Fe_2_O_3_ by ALD, and optionally depositing another TiO_2_ layer by ALD (Figure 1). The colloidal self-assembly process is performed by vertical convective self-assembly of PS particles on top of glass substrates that are immersed into PTFE beakers containing 25 mL of PS particle dispersion (0.75 mg/mL) and placed inside a humidity chamber (HCP108, Memmert) at 55 °C and 70% relative humidity for 90 h. Previously to immersion, the glass substrates were ultrasonically cleaned in 0.1 vol % aqueous mucasol solution for 1 h, brushed with mucasol solution, and rinsed with ultrapure H_2_O. The clean substrates were dried with a nitrogen stream and plasma treated using a RF plasma barrel etcher for 20 min (Polaron PT7160, VG Microtech). The resulting colloidal self-assembled PS template structures were coated with TiO_2_ by ALD in a custom-built reactor (Hamburg University of Technology, Integrated Materials Systems Group). The ALD process was operated in stop-flow mode at 95 °C and with 2 Nl/h nitrogen flow, starting after 3 h of prevacuum. TTIP as titanium precursor was heated to 85 °C, and H_2_O as oxygen precursor was kept at room temperature. During an ALD cycle, the precursors were pulsed, exposed, and purged for 1, 30, and 90 s (TTIP) and 0.2, 30, and 90 s (H_2_O), respectively, resulting in a growth per cycle (GPC) of 0.4 Å. TiO_2_ cycles were repeated until the desired coating thicknesses of 16 and 20 nm were obtained. After ALD coating, the PS templates were removed by burn-out in a muffle furnace in air, where samples were heated to 500 °C at a rate of 0.3 °C/min, kept at 500 °C for 30 min, and naturally cooled down to room temperature. The obtained TiO_2_ IOs were further functionalized with Fe_2_O_3_ in another custom-built ALD reactor (Universität Hamburg, CHyN). The Fe_2_O_3_ ALD process utilized Cp_2_Fe at 100 °C and O_3_ at room temperature (generated from O_2_ by an OzoneLab OL80W ozone generator; Ozone Services, Canada) as precursors and was operated in stop-flow mode at 200 °C. Pulse, exposure, and purge times were 2, 60, and 90 s for Cp_2_Fe and 0.08, 30, and 90 s for O_3_, respectively. The O_3_ half-cycle was twice repeated within one ALD cycle, and the GPC of Fe_2_O_3_ deposition was determined to be 0.16 Å. Fe_2_O_3_ coating thicknesses targeted 10 ALD pulses, 2 and 4 nm. To prepare TiO_2_–Fe_2_O_3_–TiO_2_ multilayer IOs, another TiO_2_ ALD process with the same parameters described above was applied. Here, TiO_2_ thicknesses of only 2 nm were deposited.

### 2.3 Structural and Optical Characterization

Microstructural characterization was conducted with a Zeiss Supra 55 VP scanning electron microscope (SEM), both in top and cross-section view, obtained after sectioning the IOs’ substrates with a glass cutter. Energy-dispersive X-ray spectroscopy (EDX) measurements were acquired with an Oxford Instruments EDX SDD detector. Optical properties were analyzed with UV/vis spectroscopy in reflection mode utilizing a Flame Extended Range Spectrometer while irradiating the samples with a deuterium-halogen light source DH-2000 (OceanOptics, Germany). Reflection measurements were conducted at normal incidence for IOs filled with air and H_2_O. Their PSB positions were analyzed with OriginPro 2021 software by applying Gaussian fits to obtain the PSB central wavelength, while the PSB edges were determined as inflection points of the PSBs and obtained from reflection data smoothed using 200 data points. X-ray diffraction patterns were obtained with a Bruker D8 Discover diffractometer. Grazing incidence diffraction (GID) configuration was used with the X-ray source fixed at an angle of 0.5°, and the detector moved along the range from 10° to 60° with a step size of 0.01° and a step time of 5 s. Phase identification was performed using commercial software from Bruker (Diffrac.EVA 5.1) and the powder diffraction file database (PDF-2 Release 2020 RDB).

### 2.4 Photocatalytic Characterization

The photocatalytic performance of TiO_2_–Fe_2_O_3_ IOs was assessed by monitoring the photocatalytic degradation of methylene blue (MB) as a model pollutant of water. A sample was mounted in a custom-built photocatalysis cell consisting of polyether ether ketone (PEEK) and a soda-lime glass window. The cell was filled with 8 mL MB solution (2.5 mg/L), which included 200 mM H_2_O_2_ and was kept in darkness for 1 h to obtain the adsorption–desorption equilibrium of molecules at the sample surface. Afterward, the cell was illuminated with UV–visible light from a Euromex LE.5211 light source equipped with a Philips 64230 FO halogen bulb and the MB absorbance was measured every 5 min. This analysis was conducted by UV–vis spectroscopy after pipetting 1 mL of the MB solution into a cuvette and placing it in the UV–vis absorbance setup consisting of a halogen light source HL-2000 (OceanOptics, Germany), glass fibers, a cuvette holder, and a Flame Extended Range Spectrometer (OceanOptics, Germany). The analyzed volume was then transferred back into the photocatalysis cell. Irradiation of the photocatalysis cell was blocked during the absorbance measurements. For further studying the MB degradation pathway, 100 mM isopropyl alcohol (IPA) as a hole scavenger was added to 8 mL MB solution (2.5 mg/L). The further processing was the same as for the H_2_O_2_-containing solution. Based on Lambert–Beer’s law, the measured MB absorbance was converted to the concentration and the photocatalytic MB degradation was examined by assuming Langmuir–Hinshelwood kinetics:^36,37^
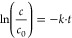
In this equation, *c* describes the concentration of the MB solution at the time *t*, *c*_0_ is the concentration at the measurement start (*t* = 0 h), and *k* denotes the apparent photocatalytic rate constant, which measures the photocatalytic activity of a sample. Unless otherwise stated, photocatalytic measurements were repeated three times for each sample to calculate the photocatalytic activity’s mean value and standard deviation. Note, the samples stayed in the same photocatalysis cell for the consecutive measurements to ensure the same positioning for all measurements.

A 400 nm long-pass filter and a 425 nm short-pass filter were installed between the light source and a sample, respectively, to assess the influence of the irradiation spectrum on the photocatalytic performance. For these measurements and the study with IPA containing solutions the samples and photocatalysis cells were new assembled. Their activity was normalized to the measured performance under the standard conditions (2.5 mg/L MB, 200 mM H_2_O_2_, full illumination spectrum) in this assembly.

## 3 Results and Discussion

### 3.1 Structural and Optical Characterization

**Figure 2:**
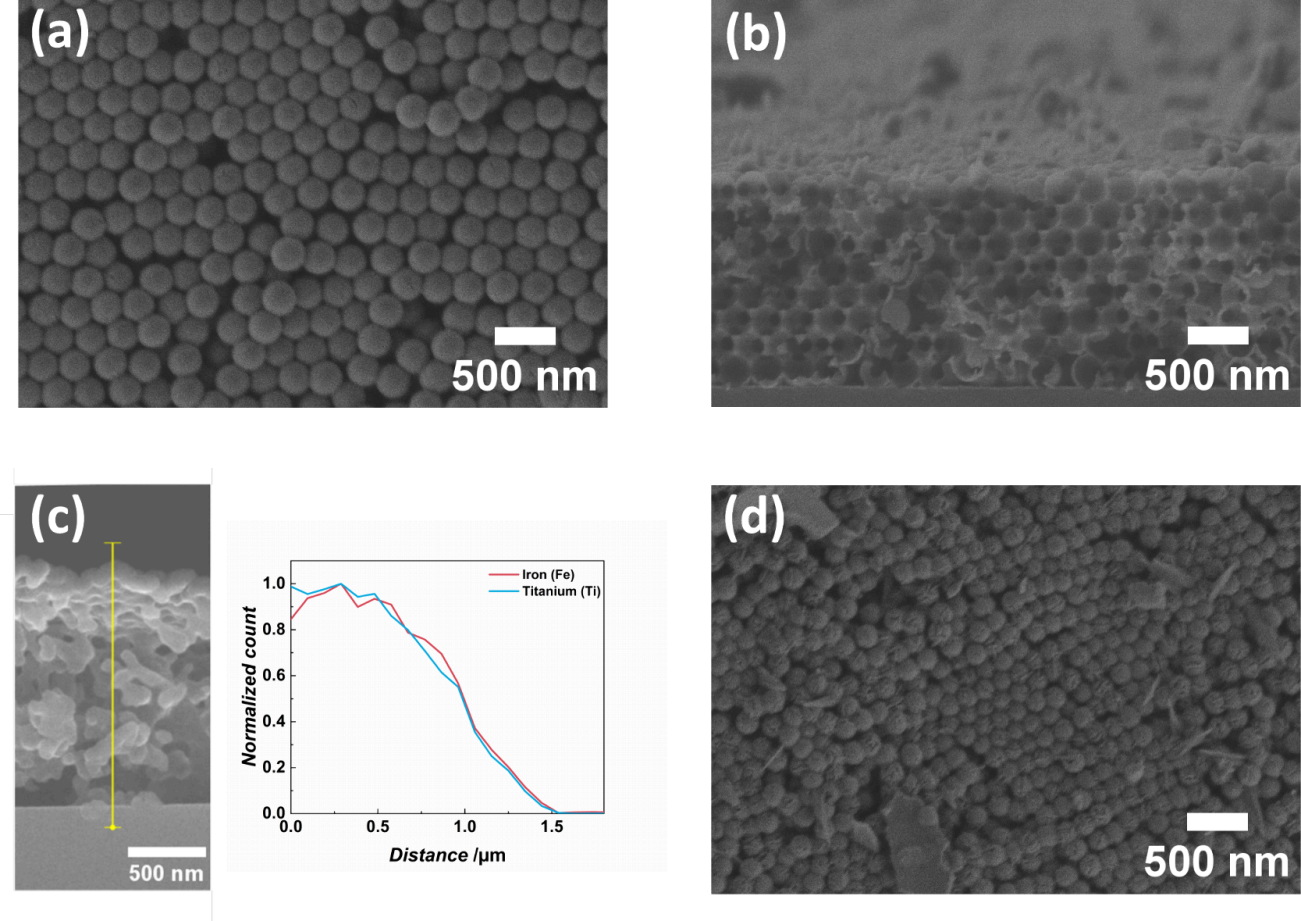
Characterization of the structural integrity and composition by SEM and EDX. (a) and (b) demonstrate the typical IO structure for (a) 16 nm TiO_2_ IO and (b) 20 nm TiO_2_–2 nm Fe_2_O_3,_ both fabricated with 252 nm PS template size. (c) an EDX scan along a 20 nm TiO_2_–2 nm Fe_2_O_3_ cross-section reveals a homogeneous distribution of iron and titanium. (d) Fe_2_O_3_-coated IOs present needle-like structures and larger particles at their top surface.

The fabricated IOs show, in general, good structural integrity, as exemplarily depicted in Figure 2. SEM images of all samples are presented in Figure S1 in the Supporting Information. Top-view and cross-section SEM images reveal a 3D PhC structure with ordered domains and hollow shells, characteristic of the IOs (Figures 2a and 2b). However, vacancies and stacking faults are also visible. These are typical defects of IO structures originating from the self-assembly of the PS particle templates.^38,39^ EDX analysis of the TiO_2_–Fe_2_O_3_ IOs demonstrates coherent signals of iron and titanium throughout the structure (Figure 2c). Since the hereby practiced ALD processes cannot produce elemental iron and titanium, these signals originate from their oxides.^35,40^ For the TiO_2_–Fe_2_O_3_ bilayer IOs, bigger flakes and needles at the top surface of the PhC are observed (Figure 2d). EDX analysis indicates that they consist of iron oxide, probably arising from the Fe_2_O_3_ ALD process as detachments from the ALD reactor walls. The template with a smaller PS particle diameter of 150 nm presents a challenge for the ALD precursor penetration and homogeneous diffusion within the 3D structure. Hence, structural defects of the IO structure are observed at some spots (Figure S1).

**Figure 3:**
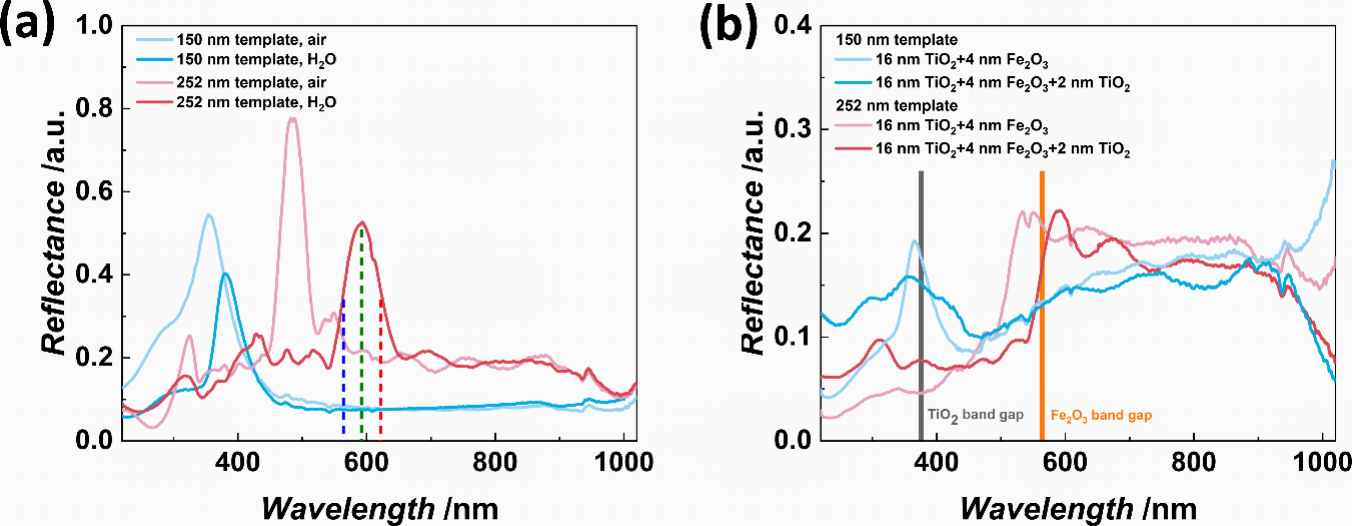
Optical properties of the prepared IOs. (a) The template size of 16 nm TiO_2_ IOs determines the PSB position, which is characterized by the PSB central wavelength (green dashed line), the PSB blue edge (blue dashed line), and the PSB red edge (red dashed line) as exemplarily shown for one measurement. Infiltrating the IOs with H_2_O redshifts the PSB due to the higher refractive index of the pore-filling medium. (b) TiO_2_–Fe_2_O_3_ IOs and TiO_2_–Fe_2_O_3_–TiO_2_ multilayer IOs with 150 nm template size feature PSBs around the electronic band gap of TiO_2_. Templates of 252 nm lead to TiO_2_–Fe_2_O_3_ IOs and TiO_2_–Fe_2_O_3_–TiO_2_ multilayer IOs with PSBs overlapping with the Fe_2_O_3_ band gap. The measurements were conducted in aqueous environment.

The fabricated IOs feature PSBs in the UV to the visible range of the electromagnetic spectrum corresponding to their structural characteristics, i.e., PS template particle size defining the template size, composition of the shell, and thicknesses of the ALD coated materials (shell thickness of the IO). Figure 3a displays the PSB positions of pure TiO_2_ IOs at normal incidence and their dependence on both the size of the PS spheres utilized as templates and the medium inside the pores. Increasing the PS particle diameter drives a redshift of the PSB position based on the increased spacing of the structure. The characterization of the optical properties not only in air, but also in an aqueous environment is crucial as the photocatalytic reactions will also take place in aqueous media and the PSB position needs to be tailored for this. Comparing the reflection spectra of TiO_2_ IOs in air to the TiO_2_ IOs filled with H_2_O reveals a PSB redshift as the refractive index of H_2_O is higher (1.33)^41^ than the refractive index of air (1.00).^42,43^ For the TiO_2_–Fe_2_O_3_ bilayer IOs, the layer composition determines their PSB position, as presented in Figure 3b. Nevertheless, only slight shifts of the PSB positions are observed for TiO_2_–Fe_2_O_3_–TiO_2_ multilayer IOs compared to TiO_2_–Fe_2_O_3_ bilayer IOs. The PSBs of all samples overlap with the respective semiconductor band gaps, i.e., TiO_2_ for 150 nm template size and Fe_2_O_3_ for 252 nm template size, as indicated in Figure 3b and Figure S2.

### 3.2 Photocatalytic Performance

#### 3.2.1 TiO_2_ and TiO_2_–Fe_2_O_3_ Inverse Opals

**Figure 4:**
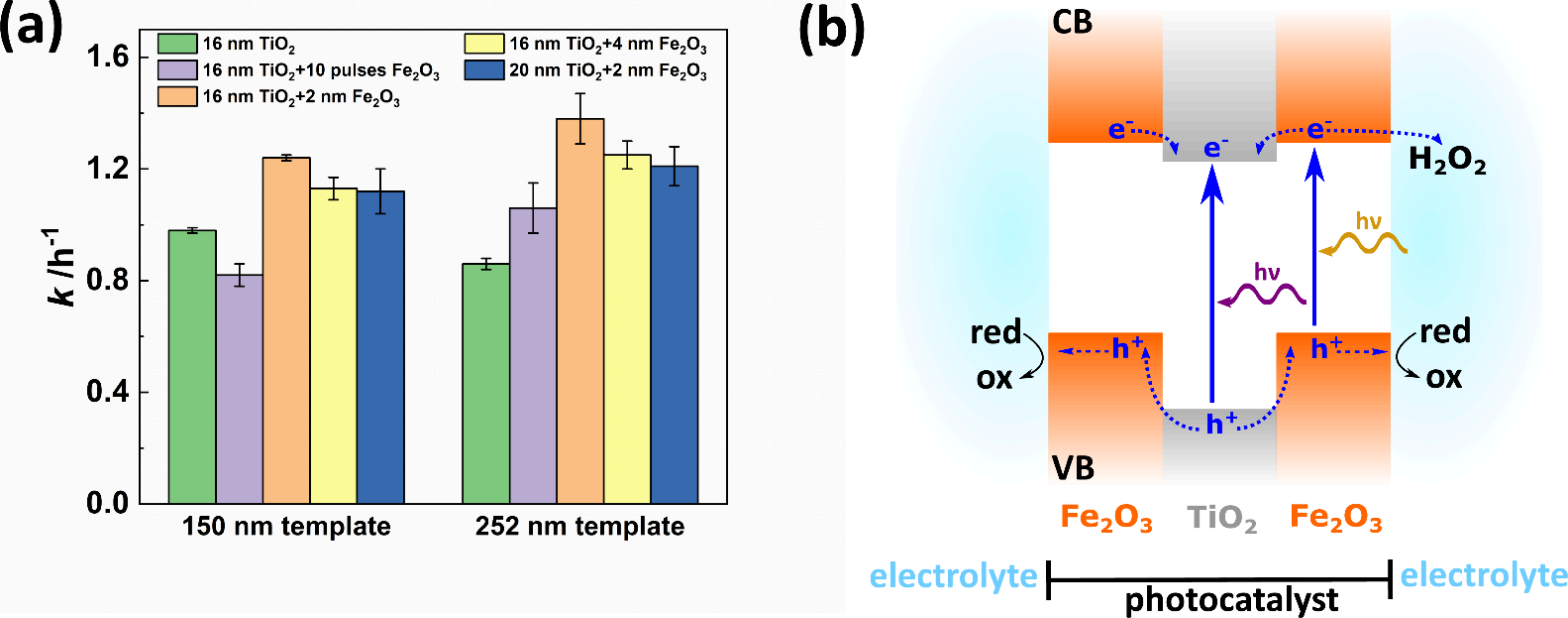
(a) The photocatalytic activity of TiO_2_–Fe_2_O_3_ bilayer IOs depends on the TiO_2_ and Fe_2_O_3_ coating thicknesses and the template size due to the alignment of the PSB with the semiconductor band gap to utilize the slow photon effect for performance enhancement. Each sample was measured three times. (b) Schematic drawing of the band structure and charge carrier movement in TiO_2_–Fe_2_O_3_ bilayer IOs. Based on the type II heterojunction, photogenerated holes inside the valence band (VB) migrate toward the Fe_2_O_3_ layers and can induce an oxidation reaction at the catalyst’s surface. Electrons in the conduction band (CB) either get inside the TiO_2_ layer or are scavenged by H_2_O_2_, which is added to the reaction solution.

Pure 16 nm TiO_2_ IOs with 150 nm template size exhibit a higher photocatalytic activity of 0.98 ± 0.01 h^–1^ than their counterparts with 252 nm template size (0.86 ± 0.02 h^–1^) due to the expected activity enhancement by the slow photon effect (Figure 4a). The individual photocatalytic activities during three consecutive measurements are shown in Figure S3 and the MB concentration decline of the individual measurements is depicted in Figure S4. The PSB blue edge of the 150 nm template size TiO_2_ IO overlaps with the band edge of TiO_2_ at around 376 nm^44^ (as illustrated in Figure 3a) and thus, the slow photon effect results in an improved photocatalytic performance. Although larger template sizes should lead to facilitated mass transfer of dye molecules into and reaction products out of the structure, this effect is overweighed by the mismatch of the PSB position concerning the TiO_2_ band gap. Hence, as expected, the slow photon effect does not enhance the photocatalytic performance in 252 nm template TiO_2_ IOs.

Additional layers of Fe_2_O_3_ significantly increase the photocatalytic activity of the IOs compared to the reference IO with only TiO_2_ (Figure 4a). This is associated with increased light absorption and facilitation of charge carrier separation. The location of the Fe_2_O_3_ band gap at ∼2.2 eV expands the absorption spectrum of the IOs to wavelengths smaller than ∼564 nm.^20,21^ Furthermore, coating Fe_2_O_3_ onto the TiO_2_ IOs results in the formation of heterojunctions at the materials interfaces, which should allow for efficient separation of the generated electron/hole pairs. The band alignment of Fe_2_O_3_ and TiO_2_ is ambiguous in literature since both type I and type II heterojunctions have been reported for such heterostructures.^22,23,25,27−29,34,45^ Accordingly, the heterojunction type depends on the fabrication method and further sample specifications, such as geometry. Based on the publications by Cao et al. and Yang et al. about ALD-based functionalization of TiO_2_ powders coated with Fe_2_O_3_ thin films and synthesis of TiO_2_–Fe_2_O_3_ thin film heterostructures, respectively, we assume that our samples feature type II heterojunctions (Figure 4b).^22,31^ Hence, photogenerated electrons move toward the conduction band (CB) of TiO_2_. At the same time, holes migrate to the valence band (VB) of Fe_2_O_3_ and can induce oxidation reactions in molecules adsorbed at the material surface. This charge carrier separation reduces the recombination of the free charge carriers and results in an improved photocatalytic performance. Free electrons can, in principle, also contribute to the degradation of organic pollutants by inducing reactions in adsorbed molecules. H_2_O_2_ was added to the reaction solution to aid the generation of radicals necessary for the photocatalytic decomposition. However, H_2_O_2_ also acts as an electron scavenger; thus, H_2_O_2_ molecules at the photocatalysts’ surface could trap free electrons.^19^ These electrons will then contribute to the photocatalytic degradation of the organic dye instead of moving toward the CB of the inner TiO_2_ layer. Although these two competing processes (electron migration to the TiO_2_ layer and electron trapping by H_2_O_2_ at the surface) cannot be clearly distinguished by the dye degradation measurements, both of them lead to a better separation of photogenerated charge carriers in the photocatalyst and hence, to an enhancement of the photocatalytic activity. Assessment of the dye degradation of the 252 nm template sample with 16 nm TiO_2_–2 nm Fe_2_O_3_ coating with a MB solution containing 100 mM IPA revealed a decrease of the activity to 26% compared to the H_2_O_2_-containing solution (Figure S5). IPA is used as hole scavenger and the decreasing activity upon its’ presence demonstrates that photogenerated holes are crucial for inducing the MB destruction in our samples.

A general increase of the photocatalytic activity for the 252 nm template compared to the smaller one is expected for TiO_2_–Fe_2_O_3_ bilayer IOs due to the slow photon effect, as in this case, the IO structural PSB was designed to match the band gap of the Fe_2_O_3_. The PSB edge of the larger template size samples overlaps with the band gap of Fe_2_O_3_, which allows for enhancement of the photocatalytic performance by the slow photon effect in Fe_2_O_3_ as observed for all TiO_2_–Fe_2_O_3_ bilayer IOs (Figure 4a).

Furthermore, the performance also depends on the Fe_2_O_3_ coating thickness. A rise of the Fe_2_O_3_ coating thickness from 10 ALD pulses to 2 nm improves the sample’ photocatalytic activity based on the material’s additional light absorption. An optimum activity of 1.38 ± 0.09 h^–1^ is demonstrated for TiO_2_–Fe_2_O_3_ bilayer IOs composed of 16 nm TiO_2_ and 2 nm Fe_2_O_3_ in comparison to 0.86 ± 0.02 h^–1^ of the single TiO_2_ IO. The photocatalytic performance is reduced when the illumination spectrum is limited to specific spectral regions (Figure S6). Specifically, the utilization of a 400 nm long-pass filter eliminates UV radiation. In this case, the photocatalytic performance of the 252 nm template sample consisting of 16 nm TiO_2_ and 4 nm Fe_2_O_3_ is reduced to 75% compared to the standard conditions. Further modification is observed when a 425 nm short-pass filter is applied. Here, the spectral range between 425 and 530 nm is suppressed and wavelengths higher than 530 nm are attenuated, while wavelengths shorter than 425 nm are transmitted without intensity alteration. With this short-pass filter, the samples’ activity decreases to 66%, demonstrating the importance of visible light radiation for inducing photocatalytic reactions by the presented heterostructure IOs. Please note that the sample was only tested once in each measurement configuration.

Nevertheless, a further increase to 4 nm Fe_2_O_3_ thickness reduces the photocatalytic activity. Although the thicker coating could absorb more light, it simultaneously reduces the gap size between neighboring shells, which might limit the diffusion of dye molecules and reaction products within the IO structure.^46^ Thus, the photocatalytic performance declines as measured for both template sizes. The higher diffusion path length for charge carriers within the 4 nm Fe_2_O_3_ coating could also result in higher charge carrier recombination rates, leading to decreasing activities with increasing thickness. Such performance decline with increasing Fe_2_O_3_ content was also observed by Pylarinou et al.^34^ for TiO_2_–Fe_2_O_3_ thin film heterostructure samples. Similar photocatalytic activities to the TiO_2_–Fe_2_O_3_ bilayer IOs of 16 nm TiO_2_ and 4 nm Fe_2_O_3_ are obtained for samples consisting of 20 nm TiO_2_ and 2 nm Fe_2_O_3_. ALD coating onto an assembled opal template structure presents a maximum coating thickness of ∼7.7% of the template sphere diameter because the tetrahedral gaps, i.e., the smallest interconnecting pores between neighboring macropores, close at this thickness.^47^ Hence, the template sizes used herein correspond to a theoretically estimated maximum coating of 11.6 and 19.4 nm for 150 and 252 nm templates, respectively. For the TiO_2_ deposition onto the opal templates, further material deposition can only occur by material transport through the octahedral gaps or at the outer surfaces of the IO, which are in contact with the environment. Since both template size IOs studied herein are coated with the same TiO_2_ thickness, the 150 nm template IO already reached the theoretical estimated maximum coating after the first ALD coating, while IOs consisting of 252 nm templates still have open tetrahedral gaps after the TiO_2_ deposition. During the Fe_2_O_3_ ALD process, the tetrahedral gaps of the 252 nm template size also get very small or even close.

Nevertheless, since the Fe_2_O_3_ coating is conducted by utilizing TiO_2_ IO structures as template instead of the PS opal, this template provides open channels between neighboring shells at the shell contact points.^39^ Thus, material diffusion through these contact points is still possible after the closure of the tetrahedral gaps by the Fe_2_O_3_ coating. Additional Fe_2_O_3_ coating may influence the charge carrier separation in the structure because the diffusion of molecules within the structure is reduced due to the tetrahedral gap closures. The slightly decreasing activities for the thicker coating, i.e., 20 nm TiO_2_ and 2 nm Fe_2_O_3_, support the assumption that diffusion limitation affects the photocatalytic properties because 2 nm Fe_2_O_3_ was the best-performing thickness for TiO_2_ IOs of 16 nm.

Functionalizing TiO_2_ IOs with Fe_2_O_3_ by ALD outperforms the photocatalytic performance of previously reported structures, namely TiO_2_ IOs modified with Fe_2_O_3_ by hydrothermal methods or chemisorption-calcination and Fe_2_O_3_-functionalized TiO_2_ nanostructures.^22,23,26,28,33,34^ This comparison considers samples of Fe_2_O_3_-coated TiO_2_ particles or inverse opals tested by photocatalytic dye degradation. Nevertheless, the exact value of the photocatalytic activity *k* depends strongly on the reaction conditions, such as illumination power, illumination spectrum, temperature, catalyst loading, type of dye, and additives in the reaction solution, which are summarized in Table S1. Hence, it is setup-specific and we, therefore, compare here the qualitative evolution of the photocatalytic activities upon Fe_2_O_3_ functionalization within publications on Fe_2_O_3_-modified TiO_2_ IOs. Our results of enhanced photocatalytic performances of TiO_2_ IOs upon functionalization with Fe_2_O_3_ agree with previous reports by Liu et al. and Pylarinou et al.^33,34^ In detail, Liu et al. observed an increase in the photocurrent density by up to 50% when TiO_2_ IOs were modified with Fe_2_O_3_ nanoparticles by the hydrothermal method. Similarly, Pylarinou et al. reported increased photocatalytic activities and photocurrent densities when they modified TiO_2_ IOs with FeO_*x*_ nanoclusters by chemisorption-calcination cycles. Moreover, they showed that the enhancement depends on the utilized iron oxide content. They attributed the maximum improvement for low iron oxide contents to the efficient charge carrier separation in combination with the utilization of the slow photon effect. High iron oxide loadings resulted in a performance decline due to increased surface recombination of photogenerated charge carriers. However, the processes involved in the photocatalytic and photoelectrochemical reaction with the structures of the two aforementioned publications differ from those in this work. Since both references functionalized TiO_2_ IOs with iron oxide particles or clusters, TiO_2_ surfaces are still in contact with the reaction solution and charges accumulated at the TiO_2_ film can induce reactions in the aqueous surrounding.

In contrast, our TiO_2_–Fe_2_O_3_ bilayer IOs prepared by ALD consist of continuously capped TiO_2_ by the Fe_2_O_3_ layers. Thus, charge transfer from the photocatalyst structure toward the solution is only possible via the Fe_2_O_3_ surfaces. To the best of our knowledge, such configuration of Fe_2_O_3_-modified TiO_2_ IOs has yet to be reported. Significant enhancement of the photocatalytic performance of TiO_2_ powder coated with Fe_2_O_3_ by ALD was reported by Cao et al.^22^ Similar to our results, they observed an optimum coating thickness of ∼2.6 nm Fe_2_O_3_ for the photocatalytic degradation of methyl orange as an organic dye. The structures formed type II heterojunctions, effectively improving the separation of photogenerated charge carriers by reducing their recombination. Further, the IOs fabricated herein present nanostructured materials that could prevent potentially hazardous leaching of photocatalytic nanoparticles into the environment.^48^ The interconnected porous structure of IOs provides a stable framework and thus, can be considered as nanostructured solids with “bulk-like” properties regarding the high stability of the structure and adhesion to the substrate during operation.^49^

#### 3.2.2 TiO_2_–Fe_2_O_3_–TiO_2_ Multilayer Inverse Opals

**Figure 5:**
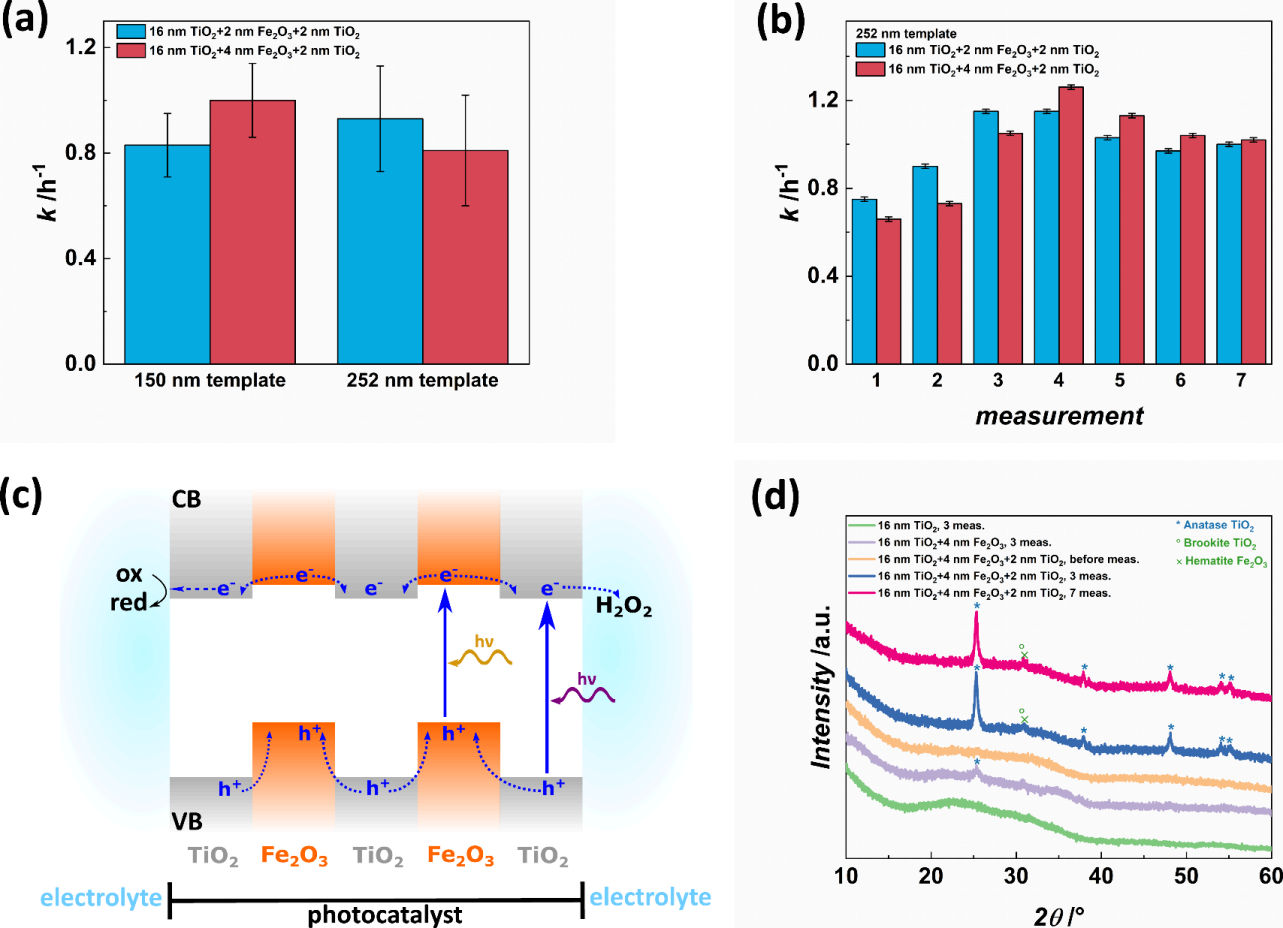
(a) The mean photocatalytic activity of TiO_2_–Fe_2_O_3_–TiO_2_ multilayer IOs after three measurements depends on the composition and template size but shows a significant standard deviation. (b) The individual activities during seven consecutive measurements of 16 nm TiO_2_–2 nm Fe_2_O_3_–2 nm TiO_2_ and 16 nm TiO_2_–4 nm Fe_2_O_3_–2 nm TiO_2_ multilayer IOs for the 252 nm template increase during the first four measurements, slightly decrease in the following two measurements and are stable afterward. (c) The band structure of TiO_2_–Fe_2_O_3_–TiO_2_ multilayer IOs depicts trapping of photogenerated holes inside the Fe_2_O_3_ layers due to adding another TiO_2_ layer. Electrons in the CB move toward the TiO_2_ layers, and those located in the outer layers can induce reductive reactions in the surrounding electrolyte or get scavenged by H_2_O_2_ molecules. (d) XRD patterns show anatase TiO_2_ peaks for Fe_2_O_3_ functionalized TiO_2_ IOs after photocatalysis measurements. Multilayer structures exhibit significantly higher peak intensities, indicating that this composition provokes photoinduced crystallization of the TiO_2_ layers.

Depositing an additional TiO_2_ thin film onto the previously presented TiO_2_–Fe_2_O_3_ IOs leads to TiO_2_–Fe_2_O_3_–TiO_2_ multilayer IOs exhibiting unstable photocatalytic activities over consecutive measurements with a reduced average performance (Figure 5a). Specifically, the activities increase within the first four measurements, then slightly decrease for two measurements, and are stable for the following trial (Figure 5b). This behavior is observed for all studied TiO_2_–Fe_2_O_3_–TiO_2_ multilayer IOs independent of the Fe_2_O_3_ coating thickness and the template size. Note, the average performance is calculated from the first three measurements to compare the multilayer IOs to the TiO_2_–Fe_2_O_3_ bilayer IOs. The multilayer IOs with 150 nm template size feature average activities of 0.83 ± 0.12 h^–1^ and 1.00 ± 0.14 h^–1^ for samples composed of 16 nm TiO_2_–2 nm Fe_2_O_3_–2 nm TiO_2_ and 16 nm TiO_2_–4 nm Fe_2_O_3_–2 nm TiO_2_, respectively. In contrast, bilayer IOs of the same template size exhibit higher activities of 1.24 ± 0.01 h^–1^ (16 nm TiO_2_–2 nm Fe_2_O_3_) and 1.13 ± 0.04 h^–1^ (16 nm TiO_2_–4 nm Fe_2_O_3_). The MB concentration decrease within the seven photocatalysis measurements of TiO_2_–Fe_2_O_3_–TiO_2_ multilayer IOs is shown in Figure S8. The individual photocatalytic performances for the 150 nm template multilayer IOs are depicted in Figure S7. Template sizes of 252 nm also result in decreased activities of 0.93 ± 0.20 h^–1^ and 0.81 ± 0.21 h^–1^ for samples consisting of 16 nm TiO_2_–2 nm Fe_2_O_3_–2 nm TiO_2_ and 16 nm TiO_2_–4 nm Fe_2_O_3_–2 nm TiO_2_, respectively, compared to the bilayer samples of this template size which show photocatalytic activities of 1.38 ± 0.09 h^–1^ for 16 nm TiO_2_–2 nm Fe_2_O_3_ and 1.25 ± 0.05 h^–1^ for 16 nm TiO_2_–4 nm Fe_2_O_3_.

These results demonstrate that the photocatalytic properties of a multilayer arrangement of TiO_2_ and Fe_2_O_3_ not depend on the thicknesses of the individual layers but rather on the template size. The TiO_2_–Fe_2_O_3_–TiO_2_ multilayer IOs exhibit lower activities than those observed for TiO_2_–Fe_2_O_3_ bilayer IOs. The significant decrease in the average photocatalytic activity of multilayer IOs after three measurements compared to the TiO_2_–Fe_2_O_3_ bilayer IOs can be attributed to two effects. First, photo-Fenton reactions at the Fe_2_O_3_ surface can no longer contribute to MB degradation because TiO_2_ overcoats Fe_2_O_3_.^1^ Second, the nonoptimal heterojunction configuration in the multilayer IOs could lead to a performance decline. Although electrons migrate toward the TiO_2_ layers and the 2 nm outer TiO_2_ film, the holes could be trapped inside the Fe_2_O_3_ layers (see schematic drawing in Figure 5c). Organic dye degradation is often mainly driven by oxidative processes involving holes, but both charge carrier types can contribute to the decomposition. The H_2_O_2_ added to the dye solution herein also acts as an electron scavenger and, therefore, could use the electrons generated in the outer TiO_2_ layers to induce photocatalytic reactions. However, electrons that migrate from the Fe_2_O_3_ layers toward the inner TiO_2_ layer and the holes trapped in the Fe_2_O_3_ layers are not available for photocatalytic reactions. Correspondingly, the photocatalytic activity is strongly reduced compared to TiO_2_–Fe_2_O_3_ bilayer IOs. IPA was utilized as hole scavenger for the 16 nm TiO_2_–4 nm Fe_2_O_3_–2 nm TiO_2_ trilayer IO to elucidate the influence of holes on the photocatalytic performance (Figure S5). With 100 mM IPA, the activity decreases to 27%, revealing that holes that migrate from the outer TiO_2_ layer to the TiO_2_ surface significantly contribute to the dye degradation in the surrounding MB solution.

#### 3.2.3 *In Situ* Photoinduced Crystallization of TiO_2_

The configuration of TiO_2_–Fe_2_O_3_–TiO_2_ multilayer IOs provokes photoinduced crystallization of TiO_2_ as characterized by XRD measurements (Figure 5d). The above-mentioned trapped holes contribute to the photoinduced crystallization of the inner TiO_2_ layer, which is enhanced by multilayer IOs (Figure 5d). While the TiO_2_ IO is still amorphous after the photocatalytic performance measurements, the TiO_2_–Fe_2_O_3_ bilayer IO with 4 nm Fe_2_O_3_ coating features a slight peak at 25° corresponding to the anatase main peak (PDF 00–064–0863). Utilizing the Scherrer equation with a shape factor of 0.9 gives an estimated average crystallite size of 9.2 nm.^50^ The TiO_2_–Fe_2_O_3_–TiO_2_ multilayer IO reveals intense peaks of the anatase phase and one peak, which indicates either brookite or hematite. The anatase (101) peak at ∼25° indicates crystallite sizes of 19.2 and 20.4 nm for the sample composed of 16 nm TiO_2_–4 nm Fe_2_O_3_–2 nm TiO_2_ after three and seven measurements, respectively. All peaks are present after three photocatalysis measurements and remain constant after seven measurements.

Moreover, the crystallite size is in the same range as the thickness of the inner TiO_2_ layer. Control experiments with Fe_2_O_3_ coated samples before the photocatalysis measurements (Figure 5d) and another control after 17 h in the reaction solution in darkness (Figure S9), i.e., the accumulated duration of 7 measurements, showed only shallow intense peaks in the XRD patterns corresponding to a crystallite size of 3.3 nm. As indicated by the crystallite sizes, crystallite growth occurs mainly within the first three measurements for which increasing activities are observed. Thus, we assume that Fe_2_O_3_ incorporation triggers the crystallization under illumination. The photoinduced crystallization is amplified for TiO_2_–Fe_2_O_3_–TiO_2_ multilayer IOs as the additional material interfaces and charge carrier trapping can facilitate the crystallization. It was previously reported that Fe ions inside the TiO_2_ lattice can create oxygen vacancies.^51,52^ These defects can serve as nucleation sites for the crystallization of the TiO_2_ film due to charge imbalances and structural distortion. In the TiO_2_–Fe_2_O_3_–TiO_2_ multilayer IOs, Fe ions present local defects in the amorphous TiO_2_ lattice at the interfaces of the TiO_2_ and Fe_2_O_3_ layers. In addition, the energy absorbed by the material during photoexcitation can also contribute to the crystallization process by providing the energy required for the crystallization.^53^ The photogenerated charge carriers can transfer energy to neighboring atoms, which can promote structural rearrangement such as crystallization.

TiO_2_ crystallization observed in this work is probably promoted by the band alignment of the individual materials. For TiO_2_–Fe_2_O_3_ bilayer IOs, the material interfaces, oxygen vacancies, and charge carriers already elicit crystallization of small parts of the TiO_2_ as indicated by the minor peak in the XRD pattern. Adding another TiO_2_ layer to the structure, i.e., the outer TiO_2_ layers in case of the TiO_2_–Fe_2_O_3_–TiO_2_ trilayer IOs, increases the number of material interfaces and confines photogenerated charge carriers to certain areas of the photocatalyst structure. Since holes migrate to the VB of Fe_2_O_3_ (Figure 5c), they are trapped inside the trilayer structure. Hence, these holes increase charge imbalances at the Fe_2_O_3_/TiO_2_ interfaces. In this way, they create additional nucleation sites for crystallization, and the required activation energy can be obtained from further photogenerated charge carriers in the material. Note, in contrast to the trilayer IOs, holes are not trapped in bilayer IO structures because they can move toward the Fe_2_O_3_ surface surrounded by the electrolyte and release their energy by inducing oxidation reactions in molecules adsorbed at the Fe_2_O_3_ surface. Both effects, the increased number of interfaces and the charge carrier trapping inside the multilayer structure are hypothesized to contribute to the strong photoinduced crystallization of the inner TiO_2_ layer in the TiO_2_–Fe_2_O_3_–TiO_2_ multilayer IOs. This photoinduced crystallization into the anatase phase improves the photocatalytic performance of the structures due to the higher inherent photocatalytic activity of anatase compared to amorphous TiO_2_.^4^ In addition, photoinduced crystallization could help to avoid shrinkage of porous structures and strong atom diffusion at interfaces, which are typical structural alterations induced by thermal crystallization.^54^ The increasing photocatalytic activities of the TiO_2_–Fe_2_O_3_–TiO_2_ multilayer IOs within the first four measurements correspond to the crystallization of the TiO_2_. The presence of oxygen vacancies, as introduced by the Fe_2_O_3_ layers, promotes charge carrier transport in TiO_2_ and improves the photocatalytic properties.^51,52,55−57^ Assuming that oxygen vacancies trigger the crystallization and are responsible for the high photocatalytic activity, a decline in their concentration would result in a reduced photocatalytic performance. Assuming that the oxygen vacancy content reaches a maximum during the crystallization process and decreases and vanishes in the final stage of the TiO_2_ crystallization, fits with the fact that the photocatalytic activity first increases and then slightly decreases until a stable performance is observed for the anatase structure. *In situ* XRD at a synchrotron during the photocatalysis characterization of the trilayer IOs could shed light on the details of the crystallization mechanism.

The emergence of photoinduced crystallization of TiO_2_ in TiO_2_–Fe_2_O_3_ multilayered structures was not previously reported. It could enable the fabrication of crystalline materials on templates unsuited for high-temperature treatments. This could, for example, be realized by incorporating ultrathin Fe_2_O_3_ layers into thicker TiO_2_ films to generate oxygen vacancies inside the complete TiO_2_ layer effectively. ALD is a commonly used technique to fabricate delta-doped structures based on self-limiting reactions. Moreover, ALD-based processing allows further combining Fe_2_O_3_-incorporated TiO_2_ with other semiconductor photocatalyst layers to separate photogenerated charge carriers as presented herein for the TiO_2_–Fe_2_O_3_ bilayer IOs. The observed photoinduced crystallization also emphasizes the influence of semiconductor heterojunctions on photocatalytic performance, structural stability, and possible tailoring. Hence, the formation of semiconductor heterostructures could further expand the application of photoinduced crystallization in various fields.^58^

## 4 Conclusion

Modifying TiO_2_ inverse opals with conformal Fe_2_O_3_ layers prepared by ALD significantly enhanced the photocatalytic properties due to additional visible light absorption and efficient separation of photogenerated charges with a photocatalytic degradation rate improvement of 27% compared to pure TiO_2_ IOs. Aligning the IOs’ PSB edge with the electronic band gap of Fe_2_O_3_ enabled further improvement of the photocatalytic performance by 60% due to the slow photon effect. Optimization of the Fe_2_O_3_ thickness resulted in a maximum activity of 1.38 ± 0.09 h^–1^ for TiO_2_–Fe_2_O_3_ bilayer IOs consisting of 16 nm TiO_2_ and 2 nm Fe_2_O_3_ coating. TiO_2_–Fe_2_O_3_–TiO_2_ multilayer IOs demonstrated reduced photocatalytic activities due to the nonoptimal band structure alignment of the individual layers. Nevertheless, the band structure provoked photoinduced crystallization of TiO_2_, resulting in an increase of the photocatalytic activity within the first four photocatalysis measurements due to anatase formation, which is known to enhance the performance compared to amorphous TiO_2_. In the future, *in situ* XRD at a synchrotron during the photocatalysis characterization could be conducted to elucidate the mechanism of photoinduced crystallization in detail. Moreover, fine-tuning the structural and optical properties of PhCs, e.g., by optimizing the IO thickness, in combination with precise adjustment of semiconductor heterostructures could further improve photocatalysts’ performance.

## Supporting Information Available

SEM images of all samples, optical properties of the prepared IOs, individual photocatalytic activities of TiO_2_ IOs and TiO_2_–Fe_2_O_3_ bilayer IOs during three consecutive measurements, dye concentration decrease during three consecutive photocatalysis measurements for TiO_2_ IOs and TiO_2_–Fe_2_O_3_ bilayer IOs, photocatalytic activities over seven measurements of multilayer IOs prepared with 150 nm template size, dye concentration decrease during seven consecutive photocatalysis measurements for TiO_2_–Fe_2_O_3_–TiO_2_ multilayer IOs, and XRD pattern of a multilayer IO exposed to the reaction solution without illumination for 17 h (PDF)
