# Supplementary material for: Enhanced Photocatalytic Properties and Photoinduced Crystallization of TiO2–Fe2O3 Inverse Opals Fabricated by Atomic Layer Deposition
Source: ACS Appl Mater Interfaces. 2024 Sep 3;16(36):46964–74. doi: 10.1021/acsami.4c10831 (PMC11403546; doi:10.1021/acsami.4c10831)
Supplement: Supplementary file 1 — am4c10831_si_001.pdf [file am4c10831_si_001.pdf]

Supporting Information

Enhanced photocatalytic properties and photo-induced crystallization of TiO_2-Fe_2O_3 inverse opals fabricated by atomic layer deposition

Carina Hedrich,[1,‡] Nithin T. James,[2,‡] Laura G. Maragno,[2] Valéria de Lima,[3] Sergio Yesid Gómez González,[3] Robert H. Blick,[1] Robert Zierold,[1,] and Kaline P. Furlan.[2,*]*

[1] Center for Hybrid Nanostructures, Universität Hamburg, 22761 Hamburg, Germany.

[2] Hamburg University of Technology (TUHH), Institute of Advanced Ceramics, Integrated Materials Systems Group, Denickestraße 15, 21073 Hamburg, Germany.

[3] Federal University of Santa Catarina (UFSC), Department of Chemical and Food Engineering (EQA), 88040-970 Florianópolis, SC, Brazil

***** Corresponding Authors: kaline.furlan@tuhh.de, rzierold@physnet.uni-hamburg.de

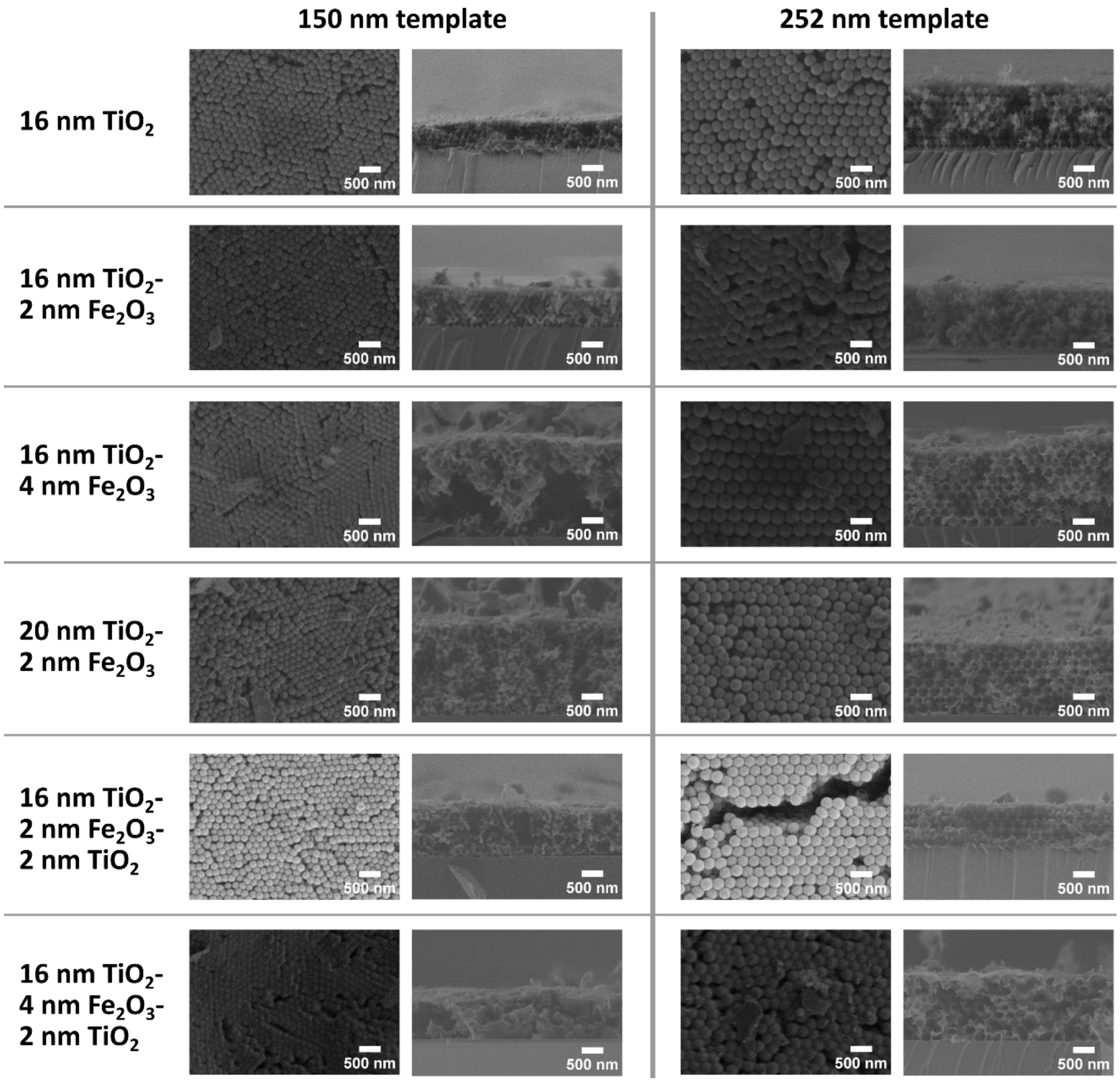

Figure S1. SEM micrographs of TiO_2-Fe_2O_3 IOs and TiO_2-Fe_2O_3 multilayer IOs fabricated with different layer thicknesses and template sizes.

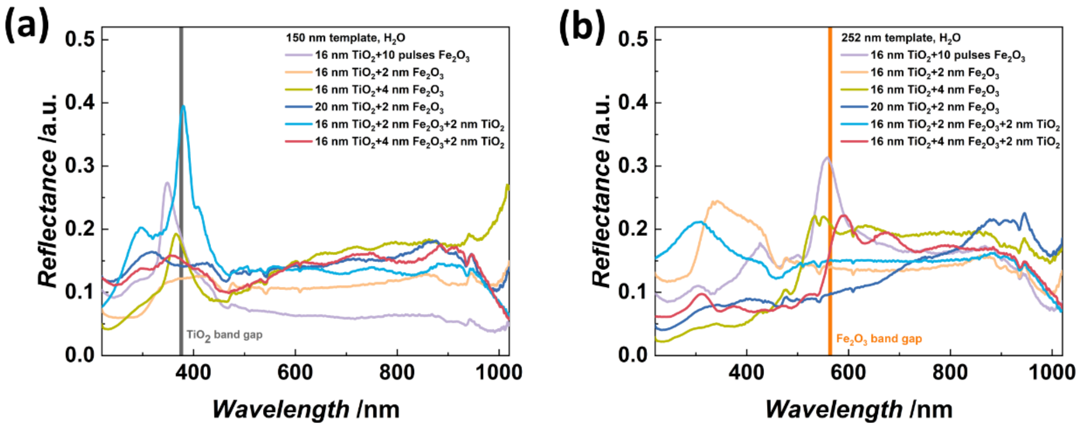

Figure S2. Optical properties of all prepared IOs measured in aqueous environment. The samples were prepared with PS particle template sizes of (a) 150 nm and (b) 252 nm.

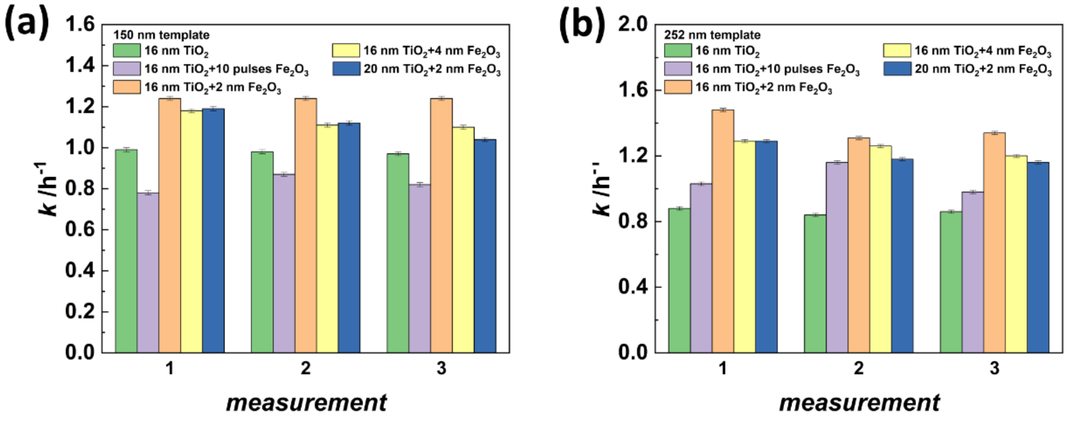

Figure S3. Individual photocatalytic activities of three consecutive measurements for TiO_2 IOs and TiO_2-Fe_2O_3 bilayer IOs. Different TiO_2 and Fe_2O_3 thicknesses were tested for template sizes of (a) 150 nm and (b) 252 nm. The

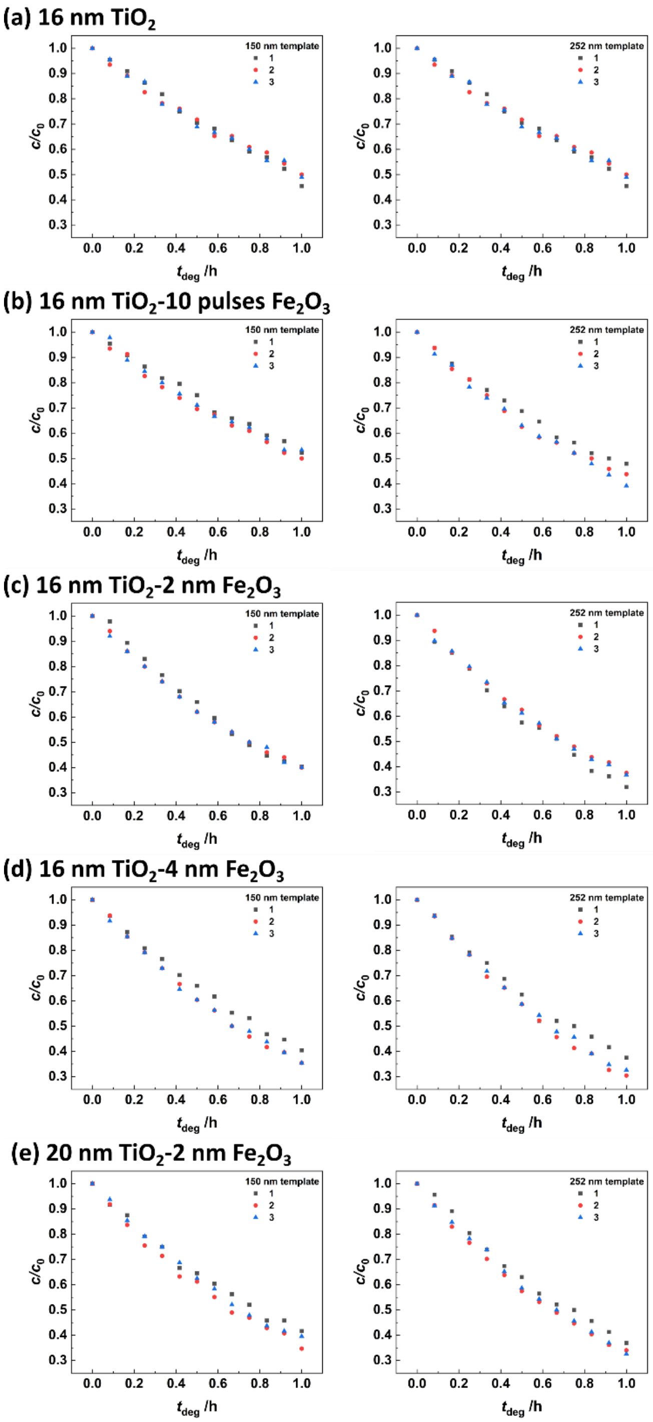

Figure S4. Dye concentration decrease during three consecutive photocatalysis measurements for TiO_2 IOs and TiO_2-Fe_2O_3 bilayer IOs. The data represents the MB degradation by (a) 16 nm TiO_2 IOs, (b) 16 nm TiO_2–10 pulses Fe_2O_3, (c) 16 nm TiO_2–2 nm Fe_2O_3, (d) 16 nm TiO_2–4 nm Fe_2O_3, and (e) 20 nm TiO_2–2 nm Fe_2O_3.

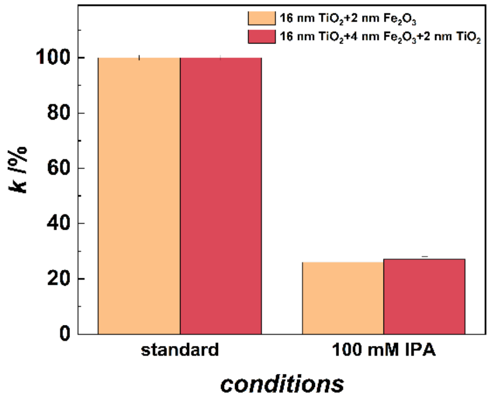

Figure S5. Assessment of the photocatalytic activities with MB solution containing 100 mM IPA as hole scavenger. Both, the 16 nm TiO_2–2 nm Fe_2O_3 bilayer IO and the 16 nm TiO_2–4 nm Fe_2O_3—2 nm TiO_2 trilayer IO demonstrate significant reduction of their photocatalytic activity compared to standard conditions. Each sample was measured three times with IPA containing solution.

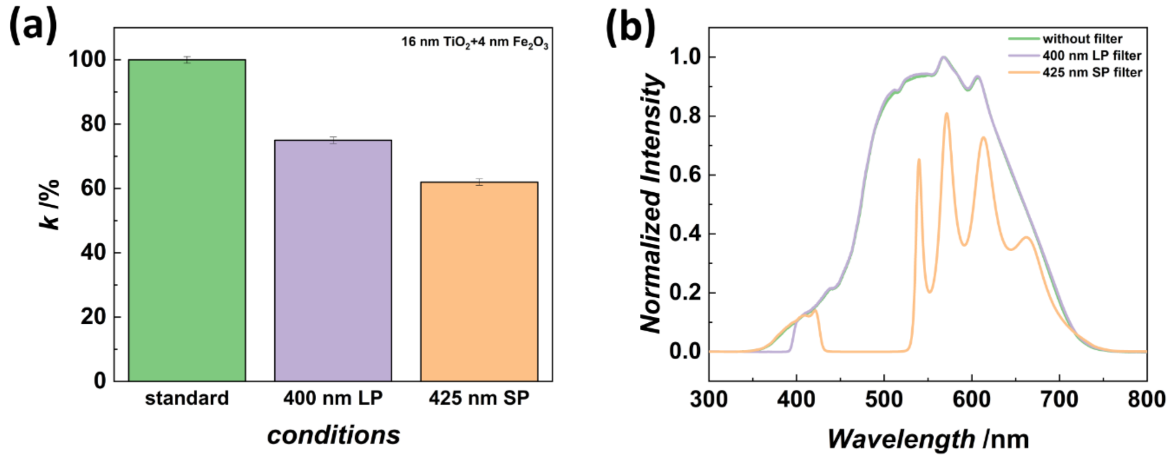

Figure S6. Photocatalytic activities under standard illumination conditions without a filter, with a 400 nm longpass (LP) filter, and with a 425 nm shortpass (SP) filter, respectively. (a) Activities of the 16 nm TiO_2–4 nm Fe_2O_3 bilayer IO normalized to the illumination without filter. The sample was measured once with each illumination spectra. (b) Optical illumination spectra of the light source with and without filters.

Table S1. Comparison of the reaction conditions and photocatalytic performances of Fe_2O_3-functionalized TiO_2 nanostructures.

Sample	Fabrication method	Organic pollutant	Illumination	Photocatalytic activity k	reference
Fe_2O_3-coated TiO_2 powder	Coating by ALD	Methyl orange 4 mg/L	300 W Xe lamp with 420 nm shortpass filter	97.4 % removal after 1.5 h	22
Fe_2O_3-coated TiO_2 nanocrystals	Hydrothermal method	Rhodamine B 50 µM, H_2O_2	8 W daylight white LED	52 % removal after 1 h	23
Fe_2O_3-coated TiO_2 nanoporous structures	ALD	PEC characterization	150 W Xe lamp		26
Fe_2O_3-coated TiO_2 microrod powder	Hydrothermal method	Orange II 20 mg/L	500 W Xe lamp with 420 nm shortpass filter	54 % removal after 3 h	28
Fe_2O_3-coated TiO_2 IO	TiO_2 IO by ALD, Fe_2O_3 coating hydrothermal method	PEC characterization	300 W Xe lamp		33
Fe_2O_3-decorated TiO_2 IO	TiO_2 IO by sol-gel method, Fe_2O_3 decoration by chemisorption calcination cycles	salicylic acid	150 W Xe lamp	~0.9 h^{-1}, 75 % removal after 1.5 h	34
TiO_2-Fe_2O_3 multilayer IOs	ALD	Methylene blue 2.5 mg/L	150 W halogen lamp	1.38 h^{-1}; 65 % removal after 1 h	This study

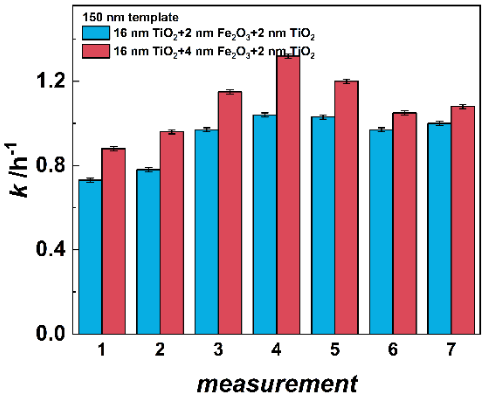

Figure S7. The individual activities during seven consecutive measurements of 150 nm template size 16 nm TiO_2–2 nm Fe_2O_3–2 nm TiO_2 and 16 nm TiO_2–4 nm Fe_2O_3–2 nm TiO_2 multilayer IOs show the same behavior as the 252 nm template size, namely increase during the first four measurements, slight decline in the following two measurements, and stable performance afterwards.

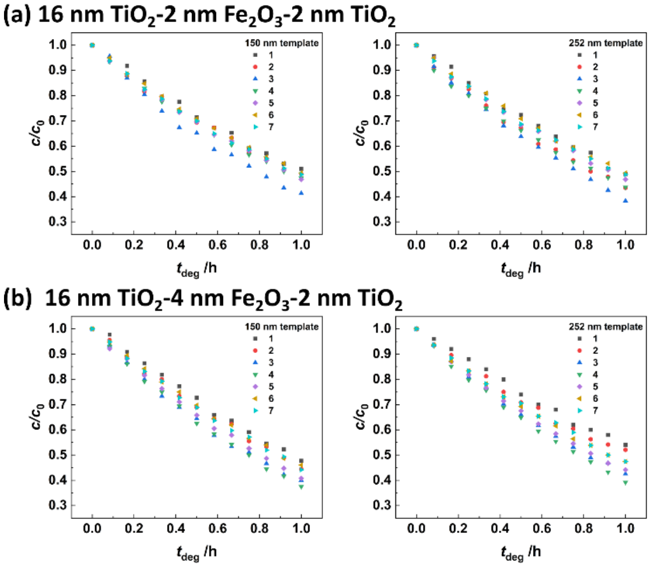

Figure S8. MB concentration decrease during seven consecutive measurements of (a) 16 nm TiO_2–2 nm Fe_2O_3–2 nm TiO_2 and (b) 16 nm TiO_2–4 nm Fe_2O_3–2 nm TiO_2 multilayer IOs.

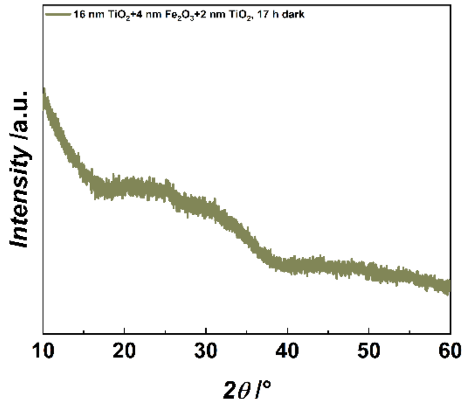

Figure S9. XRD pattern of a TiO_2-Fe_2O_3 multilayer IO composed of 16 nm TiO_2, 4 nm Fe_2O_3, and 2 nm TiO_2. The sample was kept in the reaction solution for 17 h in darkness and does not show peaks indicating crystalline TiO_2 phases.
